# Three-dimensional choroidal contour mapping in healthy population

**DOI:** 10.1038/s41598-024-56376-9

**Published:** 2024-03-14

**Authors:** Supriya Arora, Sumit Randhir Singh, Brian Rosario, Mohammed Nasar Ibrahim, Amrish Selvam, Arman Zarnegar, Sanjana Harihar, Vinisha Sant, Jose Alain Sahel, Kiran Kumar Vupparaboina, Jay Chhablani

**Affiliations:** 1 Bahamas Vision Centre and Princess Margaret Hospital, Nassau NP, Bahamas; 2 Sri Sai Lions Netralaya and Sri Sai Eye Hospital, Patna-20, Bihar India; 3 UPMC Eye Center, University of Pittsburgh, Pittsburgh, USA; 4 Department of Ophthalmology, University of Pittsburgh School of Medicine, Pittsburgh, USA

**Keywords:** Choroidal contour, Choroidal inner boundary, Choroidoscleral interface, Normative database, 3-Dimensional choroidal surface, Health care, Medical research

## Abstract

Purpose was to study 3-dimensional choroidal contour at choroidal inner boundary (CIB) and choroidal outer boundary (COB) in healthy eyes. Healthy eyes imaged on wide field swept-source optical coherence tomography were included. Delineation of CIB and COB was done based on our previously reported methods. Quantitative analysis of the surfaces of CIB and COB was based on analyzing best fit spherical radius (R) (overall and sectoral). One hundred and seven eyes of 74 subjects with a mean age of 46.4 ± 19.3 years were evaluated. Overall, R COB (mean ± SD: 22.5 ± 4.8 mm) < R CIB (32.4 ± 9.4 mm). Central sector had the least R at COB (7.2 ± 5.9 mm) as well as CIB (25.1 ± 14.3 mm) across all age groups. Regression analysis between R (CIB) and age (r = −0.31, r^2^ = 0.09) showed negative correlation (*P* < 0.001) and that between R (COB) and age was positive (r = 0.26, r^2^ = 0.07) (*P* = 0.01). To conclude, central sector is the steepest sector in comparison to all the other sectors. This is indicative of a prolate shape of choroidal contour at CIB and COB. Outer boundary of choroid is steeper than inner boundary across all age groups. However, with ageing, outer boundary becomes flatter and inner boundary becomes steeper.

## Introduction

Study on the choroid in modern day includes evaluation of cross sectional optical coherence tomography (OCT) B scans, choroidal thickness (CT) evaluation^1^ and assessment of choroidal vascularity index (CVI)^2^. Newer approaches such as en-face structural OCT^3^, choroidal vascularity mapping^4^ and 3-dimensional (3D) visualization of choroidal vessels are also being applied for detailed understanding of choroid in health and disease. All these OCT based choroidal evaluation are on the choroidal area of OCT scan and what is missing is the evaluation of choroidal topography/surface in 3 dimensions. Studies on changes in the choroidal contour will help in the better understanding of pathogenesis of various diseases such as high myopia, dome shaped maculopathy, central serous chorioretinopathy (CSCR), age related macular degeneration (AMD) and others. It will be interesting to study changes in choroidal contour affecting visual function as well as effect of treatment of diseases on choroidal contour.

In our previous study, as a proof of concept, we developed an algorithm for 3D evaluation of choroidal contour including choroidal inner boundary (CIB) and choroidal outer boundary (COB). We compared CIB and COB in healthy eyes, eyes with CSCR and AMD in a small cohort of patients and found that the choroidal contour at CIB as well as COB is the flattest in CSCR and steepest in AMD (unpublished data). Current study is aimed at implementing that algorithm on a larger cohort of healthy eyes to establish a normative database. We also aim to understand the choroidal contour changes with age among different age groups of healthy subjects. This will be important to be able to study choroidal contour in various diseases in comparison with healthy eyes.

## Methods

This retrospective study was conducted in accordance with tenets of the Declaration of Helsinki. Ethical clearance was obtained by the institutional review board of the University of Pittsburgh. Informed consent was obtained from all participants to include their retrospective data in the study. We included only healthy eyes in this study. Patients with a history of any intraocular pathology, surgery, inflammation, glaucoma and trauma were excluded. Detailed history, vision assessment, external eye examination, slit lamp examination, intraocular pressure measurement and fundus examination was done to rule out any corneal/lenticular/anterior chamber/vitreal/retinal/optic nerve/choroidal/scleral abnormality. The refractive error of the subjects and best-corrected visual acuity (BCVA) was checked. Eyes with refractive error >  ± 2 D were excluded and only subjects with BCVA better than 20/25 were included in the study. Axial length was measured using an optical biometer (IOLMaster 700, Carl Zeiss Meditec, Jena, Germany) and eyes with axial length < 21 mm or > 26 mm were excluded. Dilated imaging on wide-field swept-source optical coherence tomography (SS-OCT) 12X12 mm on the Plex Elite 9000 device (Carl Zeiss Meditec, Dublin, CA) centred on the fovea was obtained. The quality of the scan was ensured by the in-built scoring system in the swept-source optical coherence tomography (SS-OCT) machine. A score out of 10 is rewarded by the machine for every scan. Scans with scores ≥ 6 (highlighted as green) were accepted for the analysis. Only eyes with a good quality, normal scan were accepted for this study. SS-OCT scans were exported as complete 8-bit volumes. Each OCT volume comprised 1024 B-scans and the resolution of each scan was 1024 X 1536. Patients with systemic diseases such as diabetes, hypertension, impaired renal function, thyroid disorders, vascular disorders were excluded.

### Image analysis

#### Delineation of choroidal inner boundary (CIB) and choroidal outer boundary (COB)

Choroidal boundaries COB and CIB were obtained based on our previously reported methods^5^. In particular, initial CIB and COB estimate in each B scan were obtained based on residual network-based encoder-decoder deep learning architecture (ResUnet) which were subsequently stacked in 3D to perform volumetric smoothening to get the final boundary estimates. Volumetric smoothing was done to correct the abrupt deviations in boundary estimates within each B-scan and across consecutives B-scans. To smooth abrupt boundary changes across B-scans, robust locally estimated scatterplot smoothening (RLOESS)^6^ was employed and to smooth minor deformations with and across B-scans tensor voting^7,8^ was applied. This approach achieved a Dice coefficient of 97%, against manual segmentation, for both CIB and COB.

#### Methods for quantitative analysis of choroidal inner and outer boundaries (CIB and COB)

To evaluate the CIB and COB objectively, we performed quantitative analysis of the surfaces based on analyzing best-fit spherical radius (R)^9,10^. The first best-fit spherical radius is estimated for the overall surface to understand the overall curvature of the surface. The best-fit sphere radius (R) for the point is obtained using sphereFit MATLAB toolbox developed based on the least-squares regression.

$$\left( {x - a} \right)^{2} + \left( {y - b} \right)^{2} + \left( {z - c} \right)^{2} = R^{2} ,$$

where (*a*, *b*, *c*) indicates the center and *R* indicate the radius of the sphere^9^.

Subsequently, we estimated best-fit spherical radius for each sector i.e., for central, nasal, temporal, superior and inferior sector. Intuitively, flatter surface will have larger radius and vice versa.

**Figure 1:**
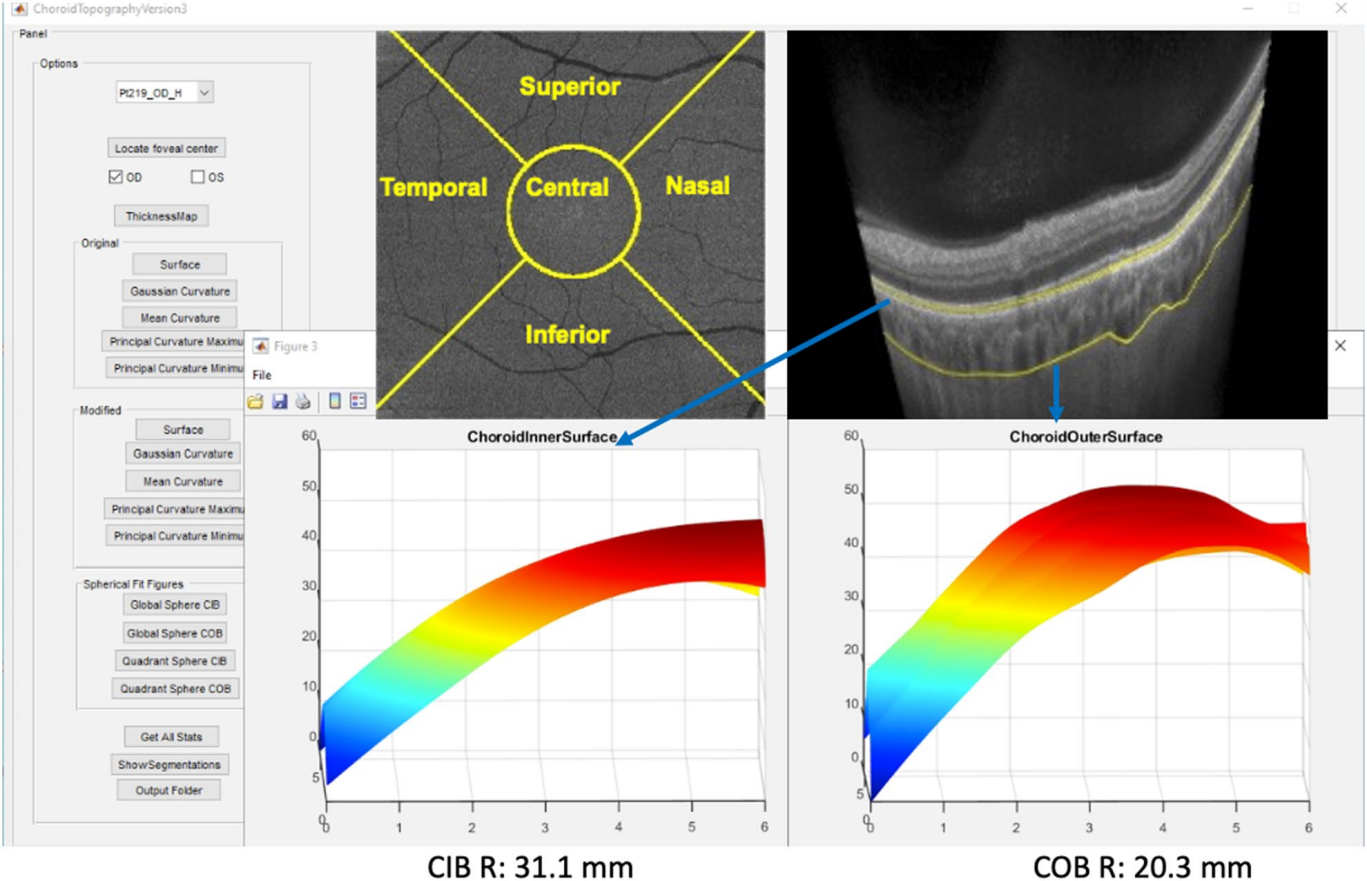
Graphical user interface (GUI) demonstrating a 3-dimensional contour of choroidal inner boundary (CIB) and choroidal outer boundary (COB) which can be rotated with the cursor to understand the shape. In this case, the best fit spherical radius of curvature (R) at COB is 20.3 mm which is lesser than CIB of 31.1 mm implicating a steeper contour at COB.

We obtained sector-wise mean and standard deviation of R to understand the curvature changes in each sector/quadrant. In particular, five quadrants—central, nasal, temporal, superior and inferior are considered centered over fovea. The center of the fovea was manually selected by the grader looking at the en-face image obtained at the internal limiting membrane (ILM) of the retina. The central quadrant is circular centered around fovea with a radius of 1 mm and the rest of the quadrants are outside the central quadrant with a 90-degree separation. We generated the binary masks of these quadrants and superimposed on the radius, thickness and curvature maps to get the quadrant wise statistics. To facilitate the expert grading and analysis, we developed an inhouse MATLAB based graphical user interface (GUI) to view the choroidal surfaces, thickness map, curvatures maps and to generate respective spread sheets consisting of sector-wise statistics. Mean values of central sector choroidal thickness were used as subfoveal choroidal thickness (SFCT) (Fig. 1).

### Statistical analysis

Data was represented as mean ± standard deviation (SD). Analysis of variance was used to compare the inner and outer choroidal spherical radius among different sectors (nasal, inferior, temporal, superior and central). Generalized estimating equation was employed to compare the choroidal parameters in different age groups (< 30, 30–44, 45–59, and ≥ 60 years). This was done as both eyes of a subset of the study subjects were included in the study. The choroidal parameters between right and left eye were compared using paired t-test. Regression analysis was used to assess the correlation between age, axial length, SFCT and radius of curvature (CIB and COB). P value ≤ 0.05 was considered statistically significant.

## Results

A total of 107 eyes of 74 subjects (including 33 subjects with bilateral eyes) were analyzed. The study cohort had a preponderance of females (42 subjects) with remaining males (32 subjects). The mean age of the study cohort was 46.4 ± 19.3 years (range, 17–89 years). The mean (± SD) BCVA (logMAR) was 0.02 ± 0.06. Axial length ranged from 22.29 to 25.94 mm (mean ± SD: 24.1 ± 1.1 mm) whereas mean SFCT was 286.0 ± 48.2 µm. One hundred and seven eyes were categorized in four categories based on age. There were 25 eyes < 30 years of age, 32 eyes in the 30–44 years age category, 23 eyes in 45–59 years of age and 27 eyes ≥ 60 years.

### Choroidal parameters

Spherical radius for CIB and COB were measured in all 5 sectors (nasal, inferior, temporal, superior and central) for the entire cohort. Moreover, spherical radius for both CIB and COB were also compared across different age groups in all sectors.

*Choroid inner boundary (CIB)*: Spherical radius was largest in inferior (mean ± SD: 37.1 ± 14.2 mm) and superior sector (32.7 ± 15.5 mm) whereas central sector had least radius of curvature (25.1 ± 14.3 mm). Overall average spherical radius for CIB was 32.4 ± 9.4 mm. Comparison of CIB within all sectors (superior, inferior, nasal, temporal and central) was statistically significant (*P* < 0.001). Spherical radius showed a declining trend as the age progressed and was evident in all sectors (Table 1). Overall mean spherical radius (CIB) across different age groups (< 30, 30–44, 45–59, and ≥ 60 years) was 35.4 ± 9.5, 33.6 ± 10.7, 32.2 ± 7.1, 28.3 ± 8.5 and the difference was statistically significant (*P* = 0.04).

**Table 1:** Showing comparison of best fit spherical radius of curvature at choroid inner boundary (CIB, mm) and best fit spherical radius of curvature at choroid outer boundary (COB, mm) across different age groups.

Descriptive	Mean ± SD (95% CI)					P value
Age	< 30	30–44	45–59	≥ 60	Total	
CIB (nasal)	32.3 ± 8.5 (28.8–35.8)	27.1 ± 10.2 (23.4–30.8)	28.0 ± 14.1 (22.0–34.1)	24.9 ± 9.9 (21.0–28.8)	28.0 ± 10.9 (25.9–30.1)	0.096
CIB (inferior)	40.8 ± 18.8 (33.0–48.5)	37.5 ± 13.0 (32.8–42.2)	39.1 ± 13.0 (33.5–44.7)	31.5 ± 10.0 (27.5–35.4)	37.1 ± 14.2 (34.4–39.8)	0.092
CIB (temporal)	35.3 ± 16.4 (28.6–42.1)	34.1 ± 12.4 (29.7–38.6)	29.8 ± 11.1 (25.0–34.5)	26.9 ± 12.1 (22.1–31.7)	31.6 ± 13.3 (29.1–34.2)	0.074
CIB (superior)	35.5 ± 20.3 (27.1–43.9)	34.7 ± 16.5 (28.7–40.6)	34.8 ± 12.1 (29.5–40.0)	26.2 ± 9.1 (22.6–29.8)	32.7 ± 15.5 (29.8–35.7)	0.086
CIB (central)	29.1 ± 14.9 (22.9–35.2)	28.6 ± 15.1 (23.1–34.0)	24.6 ± 14.5 (18.3–30.9)	17.6 ± 9.2 (13.9–21.2)	25.1 ± 14.3 (22.3–27.8)	**0.008**
CIB (overall)	35.4 ± 9.5 (31.5–39.3)	33.6 ± 10.7 (29.7–37.4)	32.2 ± 7.1 (29.1–35.2)	28.3 ± 8.5 (24.9–31.6)	32.4 ± 9.4 (30.6–34.2)	**0.04**
COB (nasal)	26.2 ± 11.8 (21.3–31.1)	23.3 ± 8.7 (20.2–26.5)	22.6 ± 10.7 (18.0–27.2)	21.6 ± 10.2 (17.6–25.7)	23.4 ± 10.3 (21.4–25.4)	0.424
COB (inferior)	19.5 ± 5.5 (17.2–21.8)	18.5 ± 4.7 (16.8–20.1)	23.3 ± 7.6 (20.0–26.6)	22.3 ± 7.3 (19.4–25.2)	20.7 ± 6.5 (19.5–22.0)	**0.017**
COB (temporal)	20.3 ± 8.7 (16.7–23.9)	17.3 ± 7.8 (14.5–20.1)	22.2 ± 6.1 (19.6–24.8)	23.4 ± 11.2 (19.0–27.9)	20.6 ± 8.9 (18.9–22.3)	**0.044**
COB (superior)	19.4 ± 10.7 (15.0–23.8)	18.2 ± 4.3 (16.7–19.7)	19.7 ± 7.1 (16.6–22.8)	20.2 ± 6.7 (17.5–22.8)	19.3 ± 7.3 (17.9–20.7)	0.759
COB (central)	7.2 ± 5.4 (5.0–9.4)	4.8 ± 3.8 (3.5–6.2)	9.0 ± 8.3 (5.4–12.6)	8.3 ± 5.3 (6.2–10.4)	7.2 ± 5.9 (6.0–8.3)	**0.037**
COB (overall)	21.5 ± 3.4 (20.1–22.9)	21.3 ± 4.2 (19.8–22.9)	23.7 ± 4.7 (21.7–25.8)	23.8 ± 6.2 (21.3–26.3)	22.5 ± 4.8 (21.6–23.4)	0.09

These values are mean radius of curvature in mm ± standard deviation (SD), (95% confidence interval (CI) is mentioned in the brackets; significant p values are highlighted in bold.

*Choroid outer boundary (COB)*: Spherical radius of curvature was largest in nasal (23.4 ± 10.3 mm) followed by inferior (20.7 ± 6.5 mm), temporal (20.6 ± 8.9 mm), superior (19.3 ± 7.3 mm) and central sectors (7.2 ± 5.9 mm). Comparison of COB across all sectors (superior, inferior, nasal, temporal and central) was statistically significant (*P* < 0.001). Overall, mean spherical radius of COB (22.5 ± 4.8 mm) was smaller compared to CIB (32.4 ± 9.4 mm). Mean COB across the various age groups was not significantly different (*P* = 0.09). Mean COB among < 30, 30–44, 45–59, and ≥ 60 years age group was 21.5 ± 3.4 mm, 21.3 ± 4.2 mm, 23.7 ± 4.7 mm and 23.8 ± 6.2 mm respectively.

Comparison of sectoral CIB and COB parameters between right and left eye (studied on 33 subjects or 66 eyes) failed to show statistically significant difference between all sectors (all *P* values ≥ 0.05) except CIB superior sector (*P* = 0.04) as shown in Table 2. Marginal model using generalized estimating equations (GEE) approach was used to compare the CIB and COB parameters in view of the repeated measurements within the same eye and to account for inter-eye correlation for both eyes. There was a statistically significant difference while comparing spherical radius of CIB and COB in different sectors (all *P* values < 0.001). This difference was also evident across different age groups (< 30, 30–44, 45–59, and ≥ 60 years) as shown in Table 3.

**Table 2:** Showing comparison of spherical radius of curvature at choroidal surfaces between right and left eye of 33 subjects (66 eyes).

Boundary	Sector	95% confidence interval		P value Sig. (2-tailed)
		Lower	Upper	
Choroidal inner boundary (CIB)	Nasal	− 7.38	2.19	0.28
	Inferior	− 6.60	2.14	0.31
	Temporal	− 3.76	4.58	0.84
	Superior	− 6.33	–0.17	**0.04**
	Central	− 7.37	2.39	0.31
	Overall (union of five sectors)	− 4.10	0.04	0.06
Choroidal outer boundary (COB)	Nasal	− 7.15	4.64	0.67
	Inferior	− 0.03	3.76	0.05
	Temporal	− 0.02	7.01	0.05
	Superior	− 3.15	1.11	0.34
	Central	− 3.02	2.80	0.94
	Overall (union of five sectors)	− 1.29	2.48	0.52

Significant p values are highlighted in bold.

**Table 3:** Shows the paired difference of spherical radius of curvature (in mm) in all the sectors (nasal, inferior, temporal, superior, central) at choroidal inner boundary (CIB) and choroidal outer boundary (COB) in different age categories.

Descriptive	Paired differences Mean ± SD (95% CI)				
Age	< 30	30–44	45–59	≥ 60	All ages (total)
Nasal (CIB vs COB)	6.1 ± 14.9 (0.0–12.2) P = 0.05	3.8 ± 11.2 (− 0.3–7.8) P = 0.066	5.5 ± 13.7 (− 0.5–11.4) P = 0.069	3.3 ± 8.3 (0.0–6.5) **P = 0.05**	4.6 ± 12.0 (2.3–6.9) **P < 0.001**
Inferior (CIB vs COB)	21.2 ± 18.7 (13.5–29.0) **P < 0.001**	19.1 ± 12.4 (14.6–23.5) **P < 0.001**	15.8 ± 11.0 (11.1–20.6) **P < 0.001**	9.2 ± 6.4 (6.7–11.8) **P < 0.001**	16.4 ± 13.4 (13.8–19.0) **P < 0.001**
Temporal (CIB vs COB)	15.0 ± 17.0 (8.0–22.1) **P < 0.001**	16.8 ± 13.0 (12.2–21.5) **P < 0.001**	7.6 ± 12.8 ( 2.0–13.1) **P = 0.01**	3.5 ± 9.9 (− 0.5–7.4) **P = **0.081	11.1 ± 14.3 ( 8.3–13.8) **P < 0.001**
Superior (CIB vs COB)	16.1 ± 12.7 (10.8–21.3) **P < 0.001**	16.5 ± 16.6 (10.5–22.4) **P < 0.001**	15.1 ± 9.7 (10.9–19.3) **P < 0.001**	6.0 ± 11.6 (1.4–10.6) **P = 0.012**	13.4 ± 13.7 (10.8–16.1) **P < 0.001**
Central (CIB vs COB)	21.9 ± 15.6 (15.4–28.3) **P < 0.001**	23.8 ± 16.0 (18.0–29.5) **P < 0.001**	15.6 ± 14.2 (9.5–21.8) **P < 0.001**	9.2 ± 7.6 (6.2–12.2) **P < 0.001**	17.9 ± 14.9 (15.1–20.7) **P < 0.001**
Overall (union of all sectors) (CIB vs COB)	14.9 ± 10.3 (10.4–19.4) **P < 0.001**	14.6 ± 10.4 (10.1–19.1) **P < 0.001**	8.4 ± 5.3 (6.1–10.7) **P < 0.001**	4.7 ± 6.1 (2.1–7.4) **P = 0.001**	9.9 ± 9.0 (8.1–11.6) **P < 0.001**

The paired difference of spherical radius of curvature (in mm) in all the sectors (nasal, inferior, temporal, superior, central) at choroidal inner boundary (CIB) and choroidal outer boundary (COB) in different age categories. It also shows overall (union of all five sectors) paired difference of spherical radius of curvature at CIB and COB.

*SD* standard deviation, *CI* confidence interval; All measurements are in mm. Significant p values are highlighted in bold.

**Figure 2:**
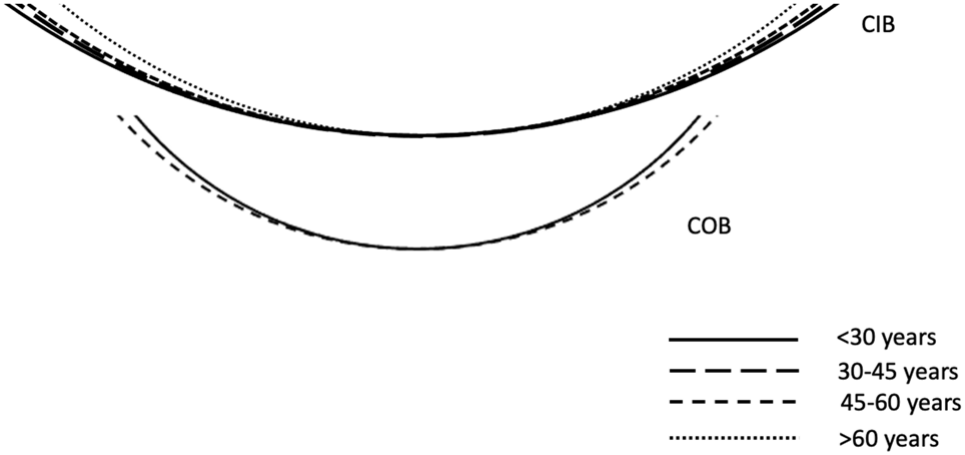
Mean of overall best fit spherical radius of curvature (R) at choroidal inner boundary (CIB) and choroidal outer boundary (COB) in different age groups. The top of the diagram demonstrates that overall CIB is becoming steeper with age. R (CIB) is 35.4 mm, 33.6 mm, 32.2 mm and 28.3 mm in age groups < 30 years, 30–44 years, 45–59 years and ≥ 60 years respectively. The bottom of the diagram shows that overall COB is becoming flatter with age. R (COB) is 21.5 mm, 21.3 mm, 23.7 mm and 23.8 mm in age groups < 30 years, 20–45 years, 45–59 years and ≥ 60 years respectively. Only 2 groups (< 30 years and 45–59 years) are shown in this diagram as the values of < 30 years and 30–44 years were very close; and the values of 45–59 years and ≥ 60 years were very close to be appreciated on the small line diagram.

Regression analysis between spherical radius (CIB) and age (r = −0.31, r^2^ = 0.09) showed negative correlation which was statistically significant (*P* < 0.001). Spherical radius of CIB reduced by 0.15 mm with each year increase in age. On the other hand, regression analysis between spherical radius (COB) and age was positive (r = 0.26, r^2^ = 0.07) which was statistically significant (*P* = 0.01). COB spherical radius increased marginally by 0.06 mm with each year increase in age (Fig. 2; Table 4). Regression analysis between R (CIB and COB) and axial length showed negative correlation (r = −0.08, r^2^ = 0.01; *P* = 0.57) and (r = −0.04, r^2^ = 0.01; *P* = 0.78) respectively. Each unit (mm) increase in axial length was associated with reduction of both spherical radius (CIB = 0.71 mm and COB = 0.20 mm). Similarly, correlation of SFCT with R of CIB (r = 0.24, r^2^ = 0.06; *P* = 0.01) and COB (r = −0.26, r^2^ = 0.07; *P* = 0.006) was also assessed. CIB (spherical radius) increased by 0.46 mm for every 10 µm increase in SFCT (*P* = 0.01). On the contrary, COB (spherical radius) showed an inverse correlation i.e., reduced by 0.27 mm for every 10 µm increase in SFCT. (Table 4) Similarly, a partial correlation of SFCT with spherical radius of CIB (*r* = 0.55, *r*^2^ = 0.30; *P* = 0.06) and COB *(*r = −0.29. *r*^2^ = 0.08; *P* = 0.36) was also assessed after adjusting for age and axial length. Multivariate regression analysis was done to assess the correlation between covariates (age, gender, axial length, SFCT) and radius of curvature (CIB and COB). The effect of gender on CIB (*r* = −0.13, *r*^2^ = 0.02; *P* = 0.27) and COB (*r* = 0.14, *r*^2^ = 0.02; *P* = 0.25) was not statistically significant.

**Table 4:** Shows changes in spherical radius of choroidal inner boundary (CIB) and choroidal outer boundary (COB) with increase in age, axial length and subfoveal choroidal thickness (SFCT).

Parameters	CIB	COB
Age	Steeper (r = −0.31, r^2^ = 0.09; p < 0.001)	Flatter (r = 0.26, r^2^ = 0.07; p = 0.01)
Axial length	Steeper (r = −0.08, r^2^ = 0.01; p = 0.57)	Steeper (r = −0.04, r^2^ = 0.01; p = 0.78)
SFCT	Flatter (r = 0.24, r^2^ = 0.06; p = 0.01)	Steeper (r = −0.26, r^2^ = 0.07; p = 0.006)

## Discussion

Using novel algorithm for choroidal contour mapping on healthy subjects, we noted a significant difference of radius of curvature between CIB and COB with R CIB > R COB. We report gradual decrease in radius of curvature in CIB and in contrary, an increase in COB with age. Our normative database show that the central sector was the steepest amongst all the sectors for both, CIB as well as COB. Assessment of correlation of SFCT with radius of curvature at COB and CIB revealed a positive correlation with R CIB and a negative correlation with R COB. We also noted an increase in axial length was correlated with a reduction in radius of curvature for both CIB and COB.

In healthy eyes, cornea has been shown to have a prolate shape i.e. central curvature is steeper than the periphery^11^. We noticed the same trend in choroidal curvature at choroidal inner boundary as well as choroidal outer boundary with center being the steepest sector. COB, i.e., choroidal curvature at the choroidoscleral interface, has been shown to have a bowl shape contour at the posterior pole in healthy eyes in previous studies^12,13^. Some authors have called this same bowl shape as convex^12^ and others have called it concave^13^ when looking from inside out. Chen et al. have shown prolate retinal shape in emmetropic eyes^14^. However, these results were not reproducible, wherein Atchison et al. showed most emmetropic eyes possess oblate shape (steepening towards the periphery)^15^. Our quantitative sectoral analysis has a robust methodology which is superior to the qualitative assessments and showed a significantly lower radius of curvature in central sector (steep center/prolate shape) versus other sectors (nasal, temporal, superior, inferior) at CIB and COB.

Myopic and hyperopic defocus is known to cause increase and decrease in choroidal thickness (CT) respectively which is both rapid and reversible upon removal of the inciting stimulus^16^. Animal models including chicks have demonstrated choroidal thinning with myopia progression which correlated well with the reduction in choroidal blood flow. The choroidal thinning however improved with the reversal of myopia^17^. Whether these changes in choroidal thickness translate into similar changes of choroidal contour is unclear at present.

On comparison of overall CIB versus COB, overall COB was steeper than CIB. On comparison of corresponding sectors, COB was still steeper than CIB in all sectors. On evaluation of relationship of age to the choroidal contour, it was found that with age choroidal contour becomes steeper at CIB and flatter at COB. This is interesting because, although overall CIB becomes steeper with age, the significant difference between COB and CIB (COB being steeper than CIB) is still maintained in all sectors and across all age groups.

On sectoral analysis of choroidal contour at CIB, the central CIB became significantly steeper with age, but CIB in temporal, nasal, superior and inferior quadrant did not change significantly with age. This shows that age related changes in choroidal contour at CIB were primarily in the central sector. Sectoral analysis of choroidal contour at COB demonstrated that flattening of contour with age was significant in inferior, temporal and central sectors.

With age, there is an increased biomechanical stiffness in the sclera. This stiffness also varies between regions with anterior sclera showing largest stiffness growth with advancing age and posterior sclera showing the least^18^. The gross shape of sclera also changes with ageing. In an OCT based study by Tun and associates, it was shown that the shape of peripapillary sclera changes as a function of age^19^. The anterior surface of sclera had a characteristic V-shape with the tip of the V pointing towards the orbit. This V shape was shown to become more prominent with age, worse vision, thinner cornea, greater axial length, lower CT in peripapillary area and deeper anterior lamina cribrosa. Changes in scleral structure as well as composition have been identified in human myopia and experimental animal myopia models^20^. With increase in axial length, scleral thickness decreases in the posterior globe segment^21^. Also, there is reduction in the collagen fibril diameter in human myopic eyes indicative of tissue remodeling with changes in axial length^22^. It is known that the mechanical stress due to distension of vitreous cavity in myopia leads to thinning and traction on the chorioretinal surface causing lacquer cracks, retinal tears, posterior staphyloma, choroidal neovascularization and other complications^23,24^. Similarly, shorter axial length in hypermetropia may cause complications such as angle closure glaucoma due to crowding of anterior segment structures^25^. Optical defocus in chick models has been shown to induce rapid changes in proteins in the retina or RPE that have previously been linked with inherited and age related ocular pathologies in humans^26^. It has been suggested that during development, choroidal shape and thickness influences the growth of sclera and length of the eye and thus play an important role in emmetropization of the eye^27^. It will be interesting to study the relationship of change in choroidal contour with various diseased states.

With increase in axial length, steepness was noted to increase at COB as well as CIB but it did not reach statistical significance. Interestingly, corneal radius of curvature increases as axial length increases i.e., cornea becomes flatter with increase in axial length^28^.

On evaluation of 33 subjects whose bilateral eyes were included in our dataset, it was shown that there was no significant difference between right eyes and left eyes in choroidal contour at CIB or COB. Previous studies on choroidal thickness have also demonstrated no significant interocular difference^29^.

The current study has certain limitations. Our sample size was small and a bigger sample size would have added more strength to the study. Only 12X12mm area of choroid was analyzed. Therefore, the peripheral choroid including the effect of vortex veins on choroidal contour was not studied. The choroidal contour especially the choroidoscleral interface does not always follow a smooth pattern and may have an inflection point or S-shaped or irregular contour^13^. Moreover, the localized effect of short posterior ciliary artery entry sites on COB was not studied. As this was a cross sectional study, we could not evaluate the long-term changes in healthy groups during follow up with age.

In conclusion, we report normative database for 3-dimensional choroidal contour mapping using novel algorithm and the changes in various age groups. In our future projects, we plan to study choroidal contour in different diseases such as high myopia, pachychoroid disease spectrum, AMD and compare with normative database.

## Data Availability

Available upon request at email: jay.chhablani@gmail.com.
